# 

**Published:** 1893-11-18

**Authors:** 


**Notice to Advertisers and Subscribers.**

All Advertisements, Orders for Copies of The Hospital, and Business Communications should be addressed to The Publisher, *not to the Editor*, The Hospital, 428, Strand, W.C.

The Cost of Subscription, post free, is as follows:—Per week, 2½d.; per quarter, 2s. 6d.; per half-year, 5s.; per annum, 8s. 6d Foreign:—Per Annum, 10s. 8d.; payable, in all cases, in advance.

**OUR NEW OFFICES,**

428, Strand, W.C., will henceforth be the Office of this Journal.

**Notices of Births, Deaths, and Marriages** are inserted in this column at a charge of 2s. 6d. for an announcement not exceeding four lines.

### NOTICE TO CORRESPONDENTS.

All MS., Letters, Books for Review, and other matters intended for the Editor should be addressed The Editor, The Lodge, Porchester Square, London, W.

The Editor cannot undertake to return rejected MS., even when accompanied by stamped directed envelope.

**"Burdett's Hospital Annual," 1893.**

Fourth year of publication. 700 pp. Boards, gilt lettering. A most complete guide to British, Colonial, Indian, and American Hospitals, Dispensaries, Nursing and Convalescent Institutions, and Asylums. Price 5s. Published by The Scientific Press (Limited), 428, Strand, W.C.

**NOTICE.—The Index for Vol. XIV. (ending Sept. 30th, 1893) is on sale at the Office of this Journal, price 2½d., post free.**

### SCRAPS AND GLEANINGS.

Mr. H. M. Stanley has contributed £50 towards the memorial hall to be erected in Carrick-on-Shannon in memory of the late Dr. Parke.

For the relief of persons affected with diseases of the throat, nose, eyes, ears, and lungs, a new hospital is in course of completion for New York City.

By the will of the late Mrs. Anne Sweetser, of Boston, 1,000 dollars are bequeathed respectively to the Free Hospital for Women and to the Massachusetts Eye and Ear Infirmary.

Mr. Passmore Edwards has generously expressed his wish to bear the entire cost of the erection of a new wing for the West Ham Hospital, the estimate for which amounts to £2,000.

Dr. Brissot is reported to have been provisionally appointed Senior Physician at the Salpetriere Hospital, in succession to the great neurologist, the late Dr. Charcot, under whom he studied.

At the quarterly meeting of the Governors of the Leicester Infirmary, the debit balance was declared to be £1,314. This sum, large as it is, is less by £300 than the debt during the same period last year.

A course of six lectures on "Sick Nursing," beginning November 15th at four p.m., will be held under the auspices of the National Health Society, by permission of the Duchess of Bedford, at 15, Belgrave Square.

Mrs. Mary T. March has bequeathed a large fortune to Washington charities. She has also directed for a new hospital to be erected, to be called "The John Pyne March Memorial Hospital," for which institution she has provided all necessary funds.

The medical missions of the Church Missionary Society are making good progress in India. The foundation stone of their hospital for women at Patna was laid recently; hospitals under the management of the society exist also at Lucknow, Benares, and Behar.

The Friendly Societies of Barrow recently held their second Hospital Sunday parade in aid of the North Lonsdale Hospital. Bad trade and the stoppage of the local steel works considerably diminished the amount received in subscriptions, but there was a large muster of men.

The Walton Local Board has agreed to admit paupers from Walton, relieved by the West Derby Guardians, to their infectious diseases hospital on payment of 20s. per week when room can be found for them by the chairman of the Health Committee and the Board's Medical officer.

The Rhyl Improvement Commissioners propose to erect an infectious diseases hospital at Morfa Villa, Towyn, at a probable cost of about £2,000. Opposition to the scheme has been based partly on the ground that the proposed site is outside the district of the Rhyl Local Board.

The Lord Mayor recently distributed a number of medals and certificates of proficiency in ambulance work amongst members of the City of London Police Force. Every officer of the force is now required to go through an ambulance class and attain a certain degree of proficiency.

The Southampton Branch of the Queen Victoria Jubilee Nurses' Institute is in such urgent need of support that unless £100 be subscribed before the end of the year the work will have to be given up. During the past twelve months 229 cases have been nursed, and 5,740 visits paid.

The financial position of the Committee of the West Riding Infirmary, Middlesbrough, is now highly satisfactory. At the recent half-yearly meeting of Governors it was announced that instead of the deficit of £158 with which the year began there was, in June, a surplus of £65 in hand.

The statement of accounts presented at the annual meeting of the Stourbridge Dispensary showed that the balance in hand from last year was £875. Improved premises for the dispensary, as has already been stated, are in course of erection, the estimate for which work is £1,443.

The highest honours at the Chicago Exhibition have just been awarded to Mellin's Food for Infants and Invalids in the form of the medal and diploma. We are glad to find that H.I.M. the Empress of Germany's testimonial and verdict on this food is confirmed by such a practical board of judges.

A concert in aid of the Heatherdene Convalescent Home was recently given at Harrogate by employés of the Corporation. Since the home was opened in September, 1892, 215 patients have been received. It is now proposed to enlarge the building sufficiently to admit men as well as women and children.

A cottage hospital was opened at Johnstone, near Paisley. It is built in the Gothic style, and stands in a good position in the midst of large grounds. The building is intended for 18 patients. Mr. C. Renshaw, M.P., the donor, has endowed it with £3,000, and promised an income besides of £100 a year for five years.

The report of the Committee of Addenbrooke's Hospital, Cambridge, shows a debit balance of £144 on this year's expenditure. It is estimated that to place the hospital on a satisfactory financial basis the income must be increased by £300 per annum. The number of indoor patients during the year has been 1,242.

The City of Dublin Hospital has been undergoing great alterations and improvements; 20 additional beds have been provided with room for as many more should sufficient funds be raised for their support, the accommodation for the staff has been much improved, and the accident ward has been placed on the ground floor.

The annual general meeting of subscribers to the Dudley Dispensary was held recently. The gross payments for the year were shown to amount to £989, while the income, including the balance brought forward, amounted to £919. The committee are hopeful that assistance will be forthcoming to clear off the adverse balance of £69.

The annual report of the Committee of the Norwood Cottage Hospital shows a small surplus on last year's expenditure, notwithstanding a decrease in subscriptions. The hospital is to be considerably enlarged. During the past year 116 patients have been treated in the institution, while many applications were refused for want of space.

Few cottage hospitals commence under such favourable circumstances as the one shortly to be erected at Bishop's Stortford. The site has been given by Sir Walter Gilbey, the cost of construction (£1,000) by Mrs. Frere and family, while £337 in donations and £71 in annual subscriptions have been promised, as well as four beds with bedding.

### BOOKS FOR NURSES.

**Ophthalmic Nursing.** An important new work on a new subject, profusely Illustrated. By SYDNEY STEPHENSON, M.B., F.R.C.S.E. With list of instruments required, glossary, &c. Crown 8vo, 200 pp. Price 3s. 6d.

**Surgical Ward-Work and Nursing.** A Practical Manual of Clinical Instruction for Nurses and Students. By ALEXANDER MILES, M.D. (Edin.), C.M., F.R.C.S.E. Over one hundred illustrations. Demy 8vo, 200 pp. Price 3s. 6d.

**Hospital Sisters and their Duties.** By EVA C. E. LÜCKES. 2nd Edition. 2s. 6d.

**The Theory and Practice of Nursing.** By PERCY G. LEWIS, M.D. 4th Edition. 3s. 6d.

**Mental Nursing.** By WILLIAM HARDING, M.B. 2s. 6d.

**Outlines of Insanity.** By FRANCIS H. WALMSLEY, M.D. Popular Edition. 1s. 6d.

**Art of Feeding the Invalid.** By a MEDICAL PRACTITIONER and a LADY PROFESSOR OF COOKERY. 3s. 6d.

**Art of Massage.** By A. CREIGHTON HALE. 70 illustrations. 6s.

**The Nurse's Dictionary of Medical Terms and Nursing Treatment.** By HONNOR MORTEN. 10th thousand in preparation.

**London: THE SCIENTIFIC PRESS (Limited), 428, Strand, W.C.**

### THE NURSES' CASE BOOK.

**FOR USE IN HOSPITAL AS WELL AS PRIVATE NURSING.**

Demy 16mo (for the apron pocket), 72pp., strong boards. Second Edition. Price 6d., or 4s. 6d. per dozen (post free).

**SPECIMEN PAGE FILLED IN.**

A Book of Tables specially prepared for use by Nurses in the Ward and in the Sick-Room. Containing Twenty-Six complete Tables, each being ruled to last one week, for keeping an exact record of a Patient's condition, including notes on Temperature, Pulse, Respiration, Motions, Age, Sex, Name, Address, Occupation, Treatment, History, Name of Medical Attendant, Number of Case, and Date of Admission.

Nurses should combine together and order parcels of over a dozen to profit by the special discount terms—*i.e.*, 4s. 6d. per dozen (post free).

**London: THE SCIENTIFIC PRESS, Ltd., 428, STRAND, W.C.**

### Books Received.

KEGAN PAUL, TRENCH, TRUBNER, AND CO. (LIMITED).

"Lectures on the Comparative Pathology of Inflammation." By Elias Metchinkoff, translated from the French by L. A. Starling and E. H, Starling, M.D.

OLIPHANT, ANDERSON, AND FERRIER.

"Little Miss Vixen." By Evelyn Everett-Green.

NICHOLS AND CO.

"Human Physiology the Basis of Sanitary and Social Science. By T. L. Nichols, M.D.

THE SCIENTIFIC PRESS.

"Ophthalmic Nursing." By Sydney Stephenson, M,B., F.R.S.C. Edin.

HORACE COX.

"Tales in Verse." By J. A. Goodchild.

BAILLIERE, TINDALL, AND COX.

"Aids to the Diagnosis and Treatment of Diseases of Children. By John McCaw.

"Cancer and Its Complications." By Charles Egerton Jennings, M.D.

SAXON AND CO.

"Everybody's Book of Acting Charades." By John J. Pledge, F.R.G.S.

**Magazines and Pamphlets.**—Public Health, Proceedings of the Society for the Study of Inebriety, Provincial Medical Journal, Edinburgh Medical Missionary Society Quarterly Paper, Action of Alcohol on the Human Body.

### MEDICAL VACANCIES AND APPOINTMENTS.

**APPOINTMENTS.**

W. J. Anderson, appointed Medical Officer for the Fillingley District of the Meriden Union. W. J. Bacque, L.R.C.P.Lond., M.R.C.S.Eng, appointed Assistant House Surgeon to the Bristol General Hospital. R. M. G. Binnie, M.D.Glasg., appointed Medical Officer of the Western District of the Durham Union. J. O. Blunden, L.R.C.S.I., L.A.H.Dub.. appointed Medical Officer for the Western District and the Workhouse of the Wirrall Union. C. W. J. Brasher, L.R.C.P.Lond., M.R.C.S.Eng., appointed Assistant House Physician to the Bristol General Hospital. W. R. Bryett, B.A.Lond., M.R.C.S., L.R.C.P., appointed Assistant House Physician to King's College Hospital. Surgeon-Lieutenant-Colonel Adye Curran, appointed Assistant Physician to St. Vincent's Hospital, Dublin. W. L. Emmerson, M.R.C.S.Eng., appointed Medical Officer for the Newfoundland District of the Leicester Provident Dispensary. E. R. Evans, appointed Medical Officer for the Llandyssil District of the Newcastle Emlyn Union. J. L. Fraser, L.D.S.R.C.S.Edin., appointed Dental Surgeon to the Northern Infirmary, Inverness. Dr. Gardiner appointed Medical Officer for the Humberstone Road District of the Leicester General Dispensary. J. F. Gordon, M.D.R.U.I., appointed Medical Officer for the Maghull District of the Ormskirk Union. C. F. Gross, L.S.A., appointed Assistant House Accoucheur to King's College Hospital. Mr. A. Hogg appointed Assistant Medical Officer to the Birmingham Parish Infirmary. J. A. Horan, M.B., B.Ch.Dub., appointed Honorary Physician to the St. John's Hospital, Limerick. R. Howden, M.B., C.M., F.R.S.Edin., appointed Professor of Anatomy in the University of Durham. W. Hunter, M.D.Edin., M.R.C.P.Lond., appointed Assistant Physician to the West London Hospital. E. Innes, M.B., C.M. Edin. Univ., appointed Medical Officer to the Nayar Brigade, Travancore, India. E. S. Jackson, M.B. C.M. Edin., reappointed Medical Officer for the Fourth District of the Lunesdale Union. Henry W. Jacob, M.A., M.D. Dub., appointed Honorary Physician to the Taunton and Somerset Hospital. N. F. Kendall, M.R.C.S.Eng., L.R.C.P.Lond., appointed Senior Resident Medical Officer to the North West London Hospital, Kentish Town Road, N.W. G. H. Loughton, M.R.C.S., LR.C.P., appointed House-Accoucheur to King's College Hospital. Mr. A. H. Minton, appointed Medical Officer for the South District of the Stratton Union. J. E. Nally, L.R.C.P., L.R.C.S.I., appointed Medical Officer for the Balla Dispensary District. R. G. Nesbitt, L.R.C.P., L.R.C.S.I., appoinied Medical Officer for the Second District of the Penzance Union. G. M. Perry, M.R.C.S., L.R.C.P., appointed House Surgeon to King's College Hospital. C. E. Russell Rendle, B.A.Oxon., M.R.C.S., L.R.C.P., appointed Assistant Surgeon to the South Devon and East Cornwall Hospital, Plymouth. Mr. Roden, appointed Medical Officer for the Keadue Dispensary District. J. F. Sarjeant, M.R.C.S.Eng., L.R.C.P.Lond., appointed Junior Resident Medical Officer to the North-West London Hospital, Kentish Town Road, N.W. J. F. W. Tatham, M.A., M.D.Dub., F.R.C.P.Edin., D.P.H.Camb., appointed Superintendent of the Statistical Department of the Registrar-General's Office, Somerset House. W. E. F. Thomson, M.A.Glasg., M.D.Edin., appointed Surgeon to the Eye Department of the Public Dispensary, Glasgow. J. Todd, M.R.C.S.Eng., reappointed Medical Officer for the Second District of the Lunesdale Union. W. Turner, M.R.C.S., L.R.C.P., L.S.A., appointed House Physician to King's College Hospital. C. Wace, M.R.C.S., L.R.C.P., appointed House Surgeon to King's College Hospital. T. Warner, M.R.C.S., L.R.C.P., appointed House Surgeon to King's College Hospital. E. E. Waters, M.B.Edin., appointed House Surgeon to the Sheffield Union Infirmary, Sheffield. R. Weekes, M.R.C.S., L.R.C.P., appointed Assistant House Surgeon to the South Devon and East Cornwall Hospital, Plymouth. J. C. Wilson, L.R.C.P., L.R.C.S.Edin., appointed Medical Officer for the Haworth District of the Keighley Union. W. Wyllie, M.D.Glasg., reappointed Medical Officer for Third District Lunesdale Union. G. B. Mower-White, M.B., B.S.Lond., F.R.C.S. Eng., appointed Surgeon to Out-patients at the Great Northern Central Hospital.

**VACANCIES.**

**Resident Medical Officers.**

Cheltenham General Hospital. Resident Surgeon for the Branch Dispensary; unmarried, or if married, without family. Salary £180 per annum, with partly-furnished house, coal, and gas. Applications to the Honorary Secretary and Treasurer by November 25th.

### The Hospital Advertiser.

### MEDICAL APPOINTMENTS.

**THE HOSPITAL FOR SICK CHILDREN,**

**INGHAM INFIRMARY AND SOUTH SHIELDS**
AND WESTOE DISPENSARY.

**THE HOSPITAL FOR SICK CHILDREN,**
GREAT ORMOND STREET, W.C.

Hospital for Sick Children, Great Ormond Street, W.C. Assistant House Surgeon, non-resident. Appointment for six months. Salary, £20. Assistant Physician; must be F. or M.R.C.P.Lond. Applications to the Secretary by November 28th.

Ingham Infirmary and South Shields and Westoe Dispensary. Junior House Surgeon; doubly qualified. Salary £50 per annum, with board and residence. Applications to the Secretary by November 20th.

Kent and Canterbury Hospital. Assistant House Surgeon; unmarried. Salary £50 per annum, with board and lodging. Applications to the Secretary by November 30th.

Liverpool Dispensaries. Assistant Surgeon; unmarried. Salary £80 per annum, with apartments, board, and attendance. Applications to R. R. Greene, Secretary, Leith Offices, 34, Moorfields, Liverpool, by November 25th.

Manchester Hospital for Consumption and Diseases of the Throat. Assistant Medical Officer; doubly qualified. Honorarium, £25 per annum. Applications to C. W. Hunt, Secretary, by November 21st.

National Sanatorium for Consumption and Diseases of the Chest, Bournemouth. Resident Medical Officer and Secretary. Salary £120 per annum, with board and lodging. Applications to the "Chairman of Committee" by December 5th.

New Hospital for Women, 144, Euston Road. Female Resident Medical Officer, and two Female Clinical Assistants for Out-patient Department. Applications to Margaret N. Bagster, Secretary, by November 30th.

Royal Free Hospital, Gray's Inn Road, W.C. Two Resident Medical Officers (House Physicians); doubly qualified. Appointment for six months, but the holder will be eligible for re-election. No salary. Board, residence, and washing provided. Applications to the Secretary by November 27th.

Royal National Hospital for Consumption, Ventnor. Assistant Resident Medical Officer; unmarried. Salary £70 per annum, with board and lodging in the hospital. Applications to be addressed to the Board of Management at the London office, 34, Craven Street, Charing Cross, W.C.

Sunderland Infirmary. House Physician; doubly qualified. Salary £80 per annum, rising £10 annually to £100, with board and residence. Applications to the Chairman of the Medical Board by December 4th.

Wolverhampton and Staffordshire General Hospital, Wolverhampton. Resident Assistant. Appointment for six months. Board, lodging, and washing provided. Applications, inscribed "Application for Resident Assistant," to the Chairman of the Medical Committee by November 30th.

### Non-Resident Medical Officers.

Ballyshannon Union (Kinlough Dispensary). Medical Officer. Salary £100 per annum, with £20 yearly as Medical Officer of Health, together with registration and vaccination fees. Applications to Mr. James McGurran, Honorary Secretary. Election on November 23rd.

Hospital of St. Peter Port, Guernsey. Surgeon. Salary £50. Applications to the President of the Poor-law Board by November 30th.

Middlesex Hospital, W. Aural Surgeon; must be F.R.C.S.Eng. Applications to F. Clare Melhado, Secretary-Superintendent, by November 23rd.

### THE EDINBURGH TRIPLE QUALIFICATION.

### Papers Recently Set.

SECOND EXAMINATION.

Questions in Advanced Anatomy. (All of which are to be answered.)

1. Describe the dissection required to expose the Posterior Tibial Artery. Give its relations and name the branches.
2. Describe the course and relations of the Glosso-Pharyngial Nerve, from the jugular foramen to its termination. Name the branches.
3. Describe the origin, course, and connections of the Thoracic Duct.
4. Describe the Prostate Gland and Prostatic portion of the Urethra under the following heads: (*a*) Position; (*b*) size and shape; (*c*) relations.

Questions in Physiology. (All of which are to be answered.)

1. Give a short sketch of the function of the different structures composing the Kidney.
2. What are the microscopic appearances and what the functions of Sympathetic Nerve Fibres?
3. Give the life history of a Blood Corpuscle. What different structures may be seen in a drop of blood?
4. What changes occur in a muscle after death?

Questions in Materia Medica. (All of which are to be answered.)

1. Mention the different Succi that are official, giving their methods of preparation and doses.
2. Give the liquid preparations of Arsenic, with their composition and doses.
3. What is the source of Asafœtida, and what preparations are made from it?

FINAL EXAMINATION.

Questions in Therapeutics. (All of which are to be answered, including the prescription.)

1. What circumstances would lead you to tap the chest in a case of acute Pleurisy with effusion?
2. How would you treat a case of Acute Pemphigus?
3. In what cases of Epilepsy would you advise the operation of Trephining?

Prescription. (To be written in unabbreviated Latin, and the directions in English.)

Prescribe a mixture containing hydrobromic acid, morphia, glycerine, syrup of orange, and infusion of gentian, for the relief of cough in an adult.

